# Strategies for utilisation management of hospital services: a systematic review of interventions

**DOI:** 10.1186/s12992-022-00835-3

**Published:** 2022-05-23

**Authors:** Leila Doshmangir, Roghayeh Khabiri, Hossein Jabbari, Morteza Arab-Zozani, Edris Kakemam, Vladimir Sergeevich Gordeev

**Affiliations:** 1 Road Traffic Injury Research Center, Tabriz University of Medical Sciences, Tabriz, Iran; 2 Department of Health Policy & Management, Tabriz Health Services Management Research Center, School of Management & Medical Informatics, Tabriz University of Medical Sciences, Tabriz, Iran; 3 Tabriz Health Services Management Research Center, Tabriz University of Medical Sciences, Tabriz, Iran; 4 Department of Community Medicine, Tabriz University of Medical Sciences, Tabriz, Iran; 5 Social Determinants of Health Research Center, Birjand University of Medical Sciences, Birjand, Iran; 6 Clinical Research Development Unit of Tabriz Valiasr Hospital, Tabriz University of Medical Sciences, Tabriz, Iran; 7 Wolfson Institute of Population Health, Queen Mary University of London, London, UK; 8 Department of Infectious Disease Epidemiology, London School of Hygiene & Tropical Medicine, London, UK

**Keywords:** Utilisation management, Utilisation review, Health policy and systems research, Hospital

## Abstract

**Background:**

To achieve efficiency and high quality in health systems, the appropriate use of hospital services is essential. We identified the initiatives intended to manage adult hospital services and reduce unnecessary hospital use among the general adult population.

**Methods:**

We systematically reviewed studies published in English using five databases (PubMed, ProQuest, Scopus, Web of Science, and MEDLINE via Ovid). We only included studies that evaluated interventions aiming to reduce the use of hospital services or emergency department, frequency of hospital admissions, length of hospital stay, or the use of diagnostic tests in a general adult population. Studies reporting no relevant outcomes or focusing on a specific patient population or children were excluded.

**Results:**

In total, 64 articles were included in the systematic review. Nine utilisation management methods were identified: care plan, case management, care coordination, utilisation review, clinical information system, physician profiling, consultation, education, and discharge planning. Primary case management was shown to effectively reduce emergency department use. Care coordination reduced 30-day post-discharge hospital readmission or emergency department visit rates. The pre-admission review program decreased elective admissions. The physician profiling, concurrent review, and discharge planning effectively reduced the length of hospital stay. Twenty three studies that evaluated costs, reported cost savings in the hospitals.

**Conclusions:**

Utilisation management interventions can decrease hospital use by improving the use of community-based health services and improving the quality of care by providing appropriate care at the right time and at the right level of care.

## Background

Hospitals provide a wide range of services necessary to meet the increasing demand for health care services and are an integral component of any health delivery system. However, inappropriate utilisation of high-cost but unnecessary or ineffective tests and medications in hospitals remains a significant challenge in many health systems [1]. Several studies documented improper hospital service use, which can be defined as “a hospital admission to provide care that could have been given in a less complex healthcare environment and at a lower cost” [2]. For example, it was previously shown that up to one-third of days of care [3–5] and diagnostic tests [6, 7], and one-fifth of all hospital admissions [8] could be inappropriate or unnecessary, negatively impacting patients’ physical and mental well-being, and driving up overall health care costs. Hence, eliminating inappropriate utilisation and waste is essential given the existing shortage of financial and human resources.

Advances in medical technology and, consequently, aggressive marketing to health care providers, direct-to-consumer advertising, political pressure from advocacy organisations, defensive medical decision making, fragmentation and discontinuity of care within and between health and social sectors - all can become the cause of healthcare overutilisation [9, 10]. Cost containment strategies can limit healthcare-related expenditure by eliminating inappropriate use of health care services while ensuring the continuous improvement of the quality of care. For example, one could consider controlling demand or supply for care, altering provision structures or hospital performance, cost-sharing, managed care, reference pricing, and generic substitution [11]. Another strategy is fostering hospital mergers and networks that may speed up restructuring through economies of scale at relatively small hospital sizes. However, creating a dominant position in the local hospital market may have an anticompetitive effect [12].

With the rising demand for healthcare services, hospitals can apply innovative methods to increase their efficiency [4]. This can be achieved by strengthening operational efficiency and targeting more significant healthcare expenditure cases. A range of measures can be used for this purpose: reducing duplication of services, decreasing the use of expensive inputs, decreasing the length of stay for inpatient care, reducing the number of long-stay beds, and reducing medical errors [13–15]. Another approach would be implementing measures that could rebalance services provision across the health system, improve allocative efficiency, and centralise administrative functions. Such measures could include shifting the provision of care from the hospital into the community, improving care coordination, strengthening preventative care, increasing the use of day surgeries, providing appropriate levels of acute care at home (hospital at home), and facilitating the discharge of patients who have to stay in hospitals longer [16, 17]. One could also consider implementing initiatives that lower management expenses and enhance administrative efficiency, such as simplifying managerial procedures; introducing uniform standards, distribution strategies and the availability of real-time consumer and provider information; improving electronic mechanisms of lodging, processing, and reimbursement of payments and claims; and outsourcing member management systems and other back-office services [18, 19].

Most importantly, besides the cost-saving and improving operational, allocative, and administrative efficiency, reducing inappropriate utilisation could eliminate potential iatrogenic effects of unnecessary services while improving healthcare quality. However, previous studies primarily focused on evaluating the effectiveness of interventions in reducing a specific service, while studies that would provide a clear overview of the utilisation management strategies for adult hospital services are still lacking. Hence, our study aimed to identify the initiatives intended to manage adult hospital services and reduce unnecessary hospital use among the general adult population.

## Methods

We conducted a systematic review of published studies investigating initiatives intended to manage adult hospital services and reduce unnecessary hospital use among the general adult population.

### Inclusion criteria

Studies were included if they reported using intervention in a general population aimed to reduce relevant primary outcomes (i.e., hospital services and/or emergency department (ED) use, frequency of hospital admissions, LOS, and use of diagnostic tests) compared to care as usual or different intervention. There were no time restrictions, but the publication language was restricted to English only.

### Exclusion criteria

We excluded studies that targeted adult patient populations only with a specific medical condition (e.g., diabetes, asthma, cardiac failure, or cancer) or children to increase homogeneity and comparability between studies.

### Search strategy

Five bibliographic databases (PubMed, ProQuest, Scopus, Web of Science, Ovid/Medline) were searched until March 2020. To capture a broad range of primary outcomes, in addition to utilisation management and utilisation review, we included the following search terms: concurrent review, prospective review, retrospective review, pre-admission review, pre-admission review, pre-certification, pre-admission certification, pre-admission certification, pre-admission authorisation, pre-admission authorisation, pre-admission testing, pre-admission testing, prior authorisation, same-day admission, physician profiling, provider profiling, physician financial incentives, demand management, case management, discharge planning, second surgical opinions, second opinions, step therapy, therapeutic substitution, closed formulary, utilisation. We additionally searched the references of included studies for other potentially essential studies.

### Study selection, data extraction, and synthesis

Results from the bibliographic databases were merged, and duplicates removed. Two reviewers (LD and RKh) independently screened the search results by title, abstract and performed a full-text review. Disagreements were resolved by discussion and consensus with a third reviewer (HJ). We extracted the following information from the studies included in the review: type of intervention, study design, details of the intervention, and effects on primary outcomes (hospital services and ED use, admissions, LOS, use of diagnostic tests) and secondary outcomes (readmissions and costs). This review follows the Preferred Reporting Items for Systematic Reviews and Meta-Analyses [20].

### Assessment of the methodological quality

We used an adapted version of the Quality Assessment Tool for Quantitative Studies (developed by the Effective Public Health Practice Project [21] to assess the methodological quality of the included studies (Appendix). The tool contains 19 items in eight key domains: (1) study design; (2) blinding; (3) representativeness in the sense of selection bias; (4) representativeness in the sense of withdrawals/drop-outs; (5) confounders; (6) data collection; (7) data analysis; and (8) reporting. Studies can have between six and eight component ratings, with each component score ranging from 1 (low risk of bias; high methodological quality) to 3 (high risk of bias; low methodological quality). An overall rating for each study was determined based on the component ratings. For example, if eight ratings have been given, a rating of ‘*strong’* was attributed to those with no weak ratings and at least five strong ratings, ‘*moderate’ to* those with one weak rating or fewer than five strong ratings, and ‘*weak’* attributed to those with two or more weak ratings. To minimise the risk of bias, assessments were completed independently by two reviewers (LD and EK). The ratings for each of the eight domains and the total rating were compared, and a consensus was reached on a final rating for each included article.

### Data Analysis

Descriptive analyses were used to describe all studies that met the inclusion criteria, focusing on study design, participants, interventions and outcomes.

## Results

**Fig. 1:**
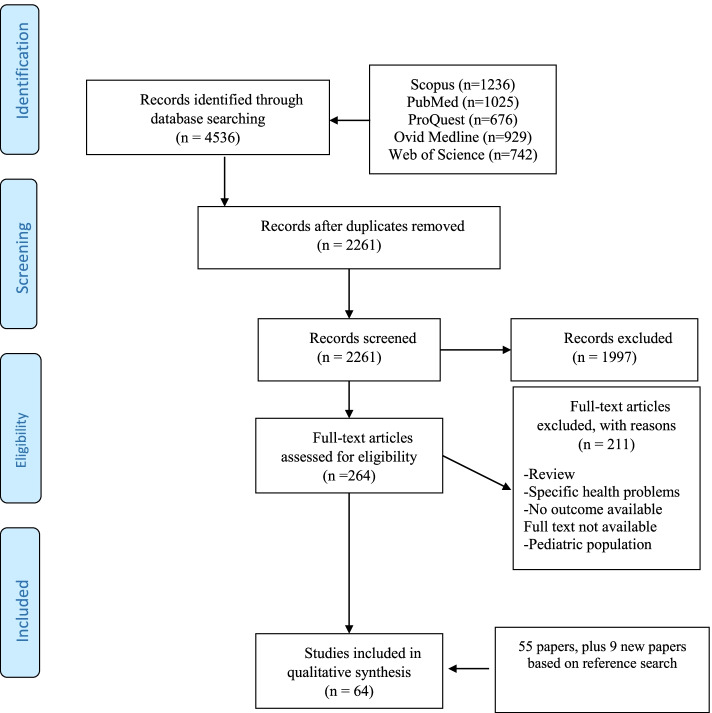
PRISMA flow diagram

The results of the screening process are shown in Fig. 1. After removing duplicates, 2261 papers were screened by title and abstract for possible inclusion in the review. The full text of 264 articles was obtained and assessed for eligibility. Of them, 56 selected papers were eligible for review. After screening references of included papers, we identified additional nine papers. Sixty four studies [22–85] met the eligibility criteria and were included in the final review.

### Characteristics of the selected studies

Included studies were published between 1982 and 2020, conducted mostly in the USA (*n* = 34) [22–24, 29–32, 37, 39, 40, 42, 43, 45, 47, 49, 56, 57, 60, 63, 65, 67–71, 73–75, 77, 78, 81, 82, 84, 85], Canada (*n* = 4) [26, 35, 55, 61], Australia (n = 4) [38, 41, 59, 83], UK (*n* = 3) [36, 64, 72], Sweden (*n* = 3) [62, 66, 76], and one each in the Netherlands [52], Korea [44], China [53], Taiwan [27], Singapore [54], and Bahrain [34]. All studies focused on the general adult population; however, some focused on specific broader subgroups with psychiatric problems [29, 45, 54, 83], comorbid conditions [49, 77], psychosocial problems (e.g., problems with housing, medical care, substance abuse, mental health disorders, or financial entitlements) [70], uninsured [30, 31, 43, 68], patients with chronic medical conditions [27, 46, 49, 61, 67], or older patients [41, 43, 47, 49, 64, 66, 67, 76]. The duration of the study follow-up ranged from one month to seven years (Table 1).

**Table 1:** Study characteristics

Author (Year) Country	Design	Health care setting	Type of intervention	Control	Health Professionals involved in an intervention	Period, months	Number of Participants
Sandberg et al. [66] (2015) Sweden	RCT	Community	Case management consisted of assessment, care coordination, providing general information, specific information and safety and monthly home visiting	Usual care	Nurse case managers, physiotherapists, physicians	12 F/U	Control: 73 Exposed: 80
Haldiman et al. [40] (2014) the United States	Cross-sectional	Hospital	Prospective review of requests for fresh –frozen plasma and platelets using guidelines and pathologists as consultants	Before review	Blood bank staff, pathologist, ordering physician	48 F/U	NR
Goodnough et al. [37] (2014) the United States	NCBA	Hospital	Concurrent review using a real-time clinical decision support system (CDSS) consisted of interruptive best practice alerts (BPAs) at the time of physician order entry (POE)	CDSS	Physicians	22 before and 30 F/U	NR
Joo [46] (2014) the United States	longitudinal	Community	Case management comprises assessment, care plans, care services in homes, clinic settings or telephone consults, evaluation	No Case Management	Nurse case managers	Up to 24 F/U	Control: - Exposed: 252
Buckley et al. [24] (2013) the United States	NCBA	Medical institution	Drug-utilization management program using evidence-based guidelines and clinical pharmacists	Pre-Implementation of Drug-Utilization Review	Clinical pharmacists, physicians, nurses, hospital administrators	6 before and 6 F/U	Control: 496 Exposed: 300
Reinius et al. [62] (2013) Sweden	RCT	Hospital	Case management using a personalised programme, telephone contact	Usual care	Nurses	12 F/U	Control: 57 Exposed: 211
Crane et al. [30] (2012) the United States	CBA	Hospital	Case management comprises drop-in group visits, telehealth line and life skills training	Before Case Management	Family physician, nurse care manager, behavioural health professional	12 before and 12 F/U	Control group: 36 Exposed: 340020
Roland et al. [64] (2012) the United Kingdom	Case-control	From hospital to community	Case management focused on integrated care, delivery system redesign, improved clinical information systems	No Case Management	Case managers, GPs, community nurses, social workers	6 before 6 F/U	Control group: 17,311 Exposed: 3646
Koehler et al. [49] (2009) the United States	RCT	Hospital	Care coordination using supplemental care bundle consists of medication counselling, reconciliation by a clinical pharmacist, patient education, enhanced discharge planning, and phone follow-up	Usual care	Care coordinator, pharmacist	2 F/U	Control: 21 Exposed: 20
Schraeder et al. [67] (2008) the United States	Quasi-experimental	Primary care	Case management emphasises collaboration between physicians, nurses and patients, risk identification, comprehensive assessment, collaborative planning, health monitoring, patient education and transitional care	Usual care	Nurse case managers, primary care physicians	36 F/U	Control: 277 Exposed: 400
Holsinger et al. [42] (2008) the United States	NCBA	Hospitals	Collaborative model of learning, a “trial-and-learn” approach to quality improvement, including Plan-Do-Study-Act cycles to test and implement changes	Before model	Physicians, medical staff, representatives from quality improvement, utilisation review or case management, billing, compliance, and medical records departments	19 before and 14 F/U	54 hospitals-
Sweeney et al. [77] (2007) the United States	Prospective cohort	HMO	Patient-centred management involves on-site assessment, education, home visits, frequent contact, and goal-oriented care plans	Usual case management	Care managers, team managers, nurses, physicians	3 to 18 F/U	Control: 398 Exposed: 358
Phillips et al. [59] (2006) Australia	NCBA	ED	Case management includes psychosocial evaluation, access to health care practitioners	Before Case Management	Nurses, allied health professionals, social workers, psychiatrists, primary care provider	12 before and 12 F/U	Control: 60 Exposed: 60
Sledge et al. [73] (2006) the United States	RCT	Primary care services	Case Management, including comprehensive medical and psychosocial assessment, care planning, follow-up, care coordination, self-management, counselling, telehealth line, home visiting	Usual care	Nurse case manager, social worker, psychiatrist, internist, primary care provider	12 F/U	Control: 49 Exposed: 47
Mahendran et al. [54] (2006) Singapore	NCBA	From hospital to community	Case Management includes care planning, care coordination, continuity of care, patient education, referral, counselling, telephone contacts, home visiting, assessment, evaluation, and supportive therapy	No Case Management	Psychiatric nurses were recruited as psychiatric case managers	12 F/U	Control: - Exposed: 227
Zemencuk et al. [85] (2006) the United States	CBA	Hospital	Physician profiling	No profiling	physicians	12 before and 12 F/U	Control: 6 hospitals Exposed:1 hospital
Latour et al. [52] (2006) the Netherlands	RCT	From hospital to community	Case management includes home visiting after discharge, assessment, set care plan consisting of psychosocial support, referral, and telephone follow up	Usual care	A nurse case manager, medical supervisor, general practitioner	6 F/U	Control: 69 Exposed: 78
Hegney et al. [41] (2006) Australia	NCBA	Hospital	Discharge planning using a risk screening tool	Before intervention	Specialist community nurse	9 before vs 9 F/U	Control: - Exposed: 2139
Horwitz et al. [43] (2005) the United States	RCT	Hospital	Case Management including referral to PCP, telephone or mail contacts, home visiting	Usual care	Case managers	6 F/U	Control:109 Exposed: 121 Control:51 Exposed: 59
Leung et al. [53] (2004) China	RCT	Community	Case Management includes regular monitoring of subjects’ health status, telehealth line, home visiting, community-based supportive services	Usual service	A nurse case manager, case geriatricians	12 F/U	Control: 47 Exposed: 45
Cox et al. [29] (2003) the United States	NCBA	Medical Center	Case management emphasises on the management of personal resources, medication compliance and therapeutic relationships	Before Case Management	Psychiatrists, nurses, psychologists, social worker	12 to 84 F/U	Control: - Exposed: 185
Hwang et al. [44] (2002) Korea	Time series	Hospital	POE system	Pre- Physician’s order entry	Physicians	3 before and 6 F/U	Control: 73 Exposed: 38
Fateha [34] (2002) Bahrain	Time series	Hospital	Concurrent Review	Before review	Medical staff	96 F/U	–
Ferrazzi et al. [35] (2001) Canada	NCBA	Community	Advanced life support drug treatment is given by ambulance attendants	Before the program	Ambulance attendants	18 before vs 18 F/U	Control: 215 Exposed: 191
Okinet al [57]. (2000) the United States	NCBA	Hospital	Case Management includes services coordination, individual and group supportive therapy, housing arrangement, financial entitlements, referral to PCP, substance abuse referral, community services, home visiting	Before Case Management	Psychiatric social worker, case manager	12 before and 12 F/U	Control: - Exposed: 53
Bates et al. [22] (1999) the United States	RCT	Hospital	Computerised physician order entry is given a reminder to the physician	No reminder	Physicians	4 F/U	Control: 5886 Exposed: 5700
Wickizer et al. [82] (1998) the United States	Retrospective analysis	Hospital	Utilisation management strategies including: Pre-admission review, concurrent review	Before Utilisation management	Nurse reviewers, physician advisers	60	49,654
Spillane et al. [74] (1997) United States	RCT	Hospital	Case management includes individualised care plans, psychosocial evaluation, care coordination	Usual care	ED physician, social worker, psychiatrist, ED nurse practitioner	12 before and 12 F/U	Control: 25 Exposed: 27
Bree et al. [23] (1996) the United States	RCT	Hospital	Pre-certification includes mandatory radiology consultation; each radiology examination requires approval by the attending radiologist before it is performed	No Pre-certification	Attending radiology consultant, radiology clerical personnel	12 F/U	Control: 1178 Exposed: 1022
Shea et al. [69] (1995) the United States	RCT	Hospital	Clinical information systems include: computer-generated informational messages directed to physicians	No message	Physicians	23 F/U	Control: 6990 Exposed: 7109
Cardiff et al. [26] (1995) Canada	Time-series	Hospitals	Utilisation management strategy includes identifying patients who did not need to be in acute care beds, as defined by the ISD-A explicit criteria and modifying the level of care for such patients	Before Utilisation management program	Nurse reviewers, physicians	12 before and 12 F/U	Control: Hospital C: 281 Hospital D: 312 Exposed: Hospital A: 600 Hospital B: 597
Styrborn [76] (1995) Sweden	Multicenter controlled trial	From hospital to community	Discharge Planning comprised: patient assessment, development of discharge plan, implementation in the form of provision of services, including patient/family education and service referrals, follow up/ evaluation	Ordinary discharge routines	Consultant geriatrician, nurse	3 F/U	Control: Hospital B: 166 Hospital C: 190 Exposed: Hospital A: 180
Rosenberg et al. [65] (1995) the United States	Case-control	Hospital	Utilisation review, second opinion, discharge planning, case management	Sham review	Nurses, physicians	8 F/U	Control: 3743 Exposed: 3702
Jambunathan et al. [45] (1995) the United States	Cross-sectional	Outpatient clinic	Case management including biopsychosocial assessment, care planning, care delivery, care coordination	Before Case Management	Nurses	18 F/U	Control: -Exposed: 21
Williams et al. [83] (1994) Australia	Cross-sectional	Hospital	Drug utilisation review	No review	Drug use review panel	Patient admission to discharge	Control: - Exposed: 75
Wickizer [81] (1992) the United States	Retrospective analysis	Hospital	Utilisation Review consists of pre-admission authorisation and concurrent review	No Review	Registered nurses, physician advisors, medical personnel	36 F/U	Control: - Exposed: 1844
Woodside et al. [84] (1991) the United States	Case-control	Hospital	Utilisation management strategies including concurrent review, consultation, discharge planning, care coordination	No Utilisation management	Care coordinator, physician, nurses	3 F/U	Control: 191 Exposed: 73
Silver et al .[71] (1992) the United States	Cross-sectional	Hospital	Prospective review using guidelines	No review	Transfusion service technical personnel, physicians	12 F/U	Control: -Exposed: 543
Fowkes et al. [36] (1986) the United Kingdom	Multicenter controlled trial	Hospitals	Appointment of a utilisation review committee, informational feedback given to physicians, the introduction of a new chest X-ray request form, concurrent review	No review	Physicians, clerical staff	12 F/U	44,632
Echols et al. [32] (1984) the United States	NCBA	Hospital	Drug utilisation Review using an antibiotic order form	Before the introduction of the order form	Physicians	25 F/U	NR
Restuccia [63] (1982) the United States	Multicenter controlled trial	Hospitals	Utilisation review consists of providing concurrent feedback to physicians	No feedback	Nurses review coordinators, physicians	2 F/U	Control: hospital D: 51 Exposed: hospital A: 145 hospital B:68 hospital C: 60
Murphy [56] (2014) the United States	NCBA	Hospital	Case management includes multidisciplinary ED care coordination, individualised ED care guidelines, and information system	Before ED-care-coordination program	Physicians, nurses, mental health and substance abuse professionals, ED nurse managers, a pharmacist, a social worker, a chaplain	12 before and 12 F/U	Control: 65 Exposed: 65
Chiang et al. [27] (2014) Taiwan	NCBA	Hospital	Case management using dynamic, internet-mediated, team-based support led by emergency physicians	Before Case Management	ED physicians, primary care physicians, psychiatrists, social workers, and pharmacologists	6 before and 6 F/U	Control: - Exposed: 14
Pillow et al. [60] (2013) the United States	NCBA	Hospital	Care plans include social work assessment, directives to call pain team for the development of pain contract, radiologic studies, out-patient referral for speciality clinics, urinary toxicology studies, managed care referral, and psychiatric assessment	Before CP	Social workers, case managers, physicians	6 before and 11 F/Uphil	Control: - Exposed: 50
Dehaven et al .[31] (2012) the United States	Quasi-experimental	From hospital to community	A community-based partnership includes improving access to a primary care provider through in-person or telephone access to the community health worker, referral	Usual care	Primary care providers, hospital-based coordinators, community health worker	12 F/U	Control: 309 Exposed: 265
Tadros et al .[78] (2012) the United States	NCBA	EMS	Case management includes coordination of treatment and social services, in-person contact, EMS interface, referrals, phone calls, transports	Before Case Management	Primary care physicians, social workers, case managers and adult protective services personnel	16 before and 15 F/U	Control: - Exposed: 51
Shah et al .[68] (2011) the United States	CBA	Primary care services	Care management includes access to medical and social resources, scheduling primary care appointments, following up on referrals, arranging for support services, e.g., housing, care transitions while in hospital, care navigation and care coordination between specialists and primary care providers	Before Case Management	Case managers, Primary care providers	12 before and 3 to 12 F/U	Control: 160 Exposed: 98
Stokes-Buzzelli S et al. [75] (2010) the United States	NCBA	Hospital	Health Information Technologies consist of identifying the most frequently presenting patients and creating individualised care plans for those patients and access to care plans through electronic medical records	No HIT	ED attending, ED medical social worker, ED mental health social worker, ED psychologist, ED resident, ED clinical nurse specialists	Same pre-and post-intervention time for each patient but varied between patients from 3 to 23	Control: - Exposed: 36
Grimmer-Somers et al. [38] (2010) Australia	NCBA	Community	Individualised care plan including health assessment, social support, problem-solving, empowerment, education, goal setting and mentoring	Before program	Social workers, nurses	12 before and 12 F/U	Control: -Exposed: 37
Grover et al. [39] (2010) the United States	NCBA	Hospital	Case management using patient care plans consisted of referral to PCP, limiting narcotic use, pain management, chemical dependency behavioural health evaluation, social services	Before Case Management	Physicians, nurses, social service providers, pain management clinicians, specialists in behavioural health	6 before and 6 F/U	Control: 96 Exposed: 96
Skinner et al. [72] (2009) the United Kingdom	CBA	Hospital	Case management includes evaluation, individualised care plan, referrals to other services, key contact, close observation	Before Case Management	ED consultant, ED specialist registrar, psychiatric nurse specialist, social workers, housing officers	6 before vs 6 F/U	Control: 21 Exposed: 36
Shumway et al. [70] (2008) the United States	RCT	Hospital	Case management including individual assessment, crisis intervention, individual and group supportive therapy, arrangement of stable housing and financial entitlements, linkage to medical care providers, referral to substance abuse services, ongoing assertive community outreach	Usual care	Psychiatric social workers, nurse practitioners, primary care physicians, psychiatrist	24 F/U	Control: 85 Exposed: 167
Pope et al. [61] (2000) Canada	NCBA	Hospital	Case management includes individualised care plan, limiting narcotics and benzodiazepines prescriptions and laboratory tests requested in ED, referral to PCP, pain program, addiction counselling, communicating care plans with other EDs, supportive therapy, arrangement of food services	Before Case management	Social workers, ED medical director, director of continuous quality improvement, patient care manager, psychiatric nurse, clinical nurse specialist, family physicians, community care providers	12 before and 12 F/U	Control: 24 Exposed: 24
Moher et al. [55] (1992) Canada	RCT	Clinical teaching units	Discharge planning based on individual patient needs	Standard medical care	Nurse	4 F/U	Control: 131 Exposed: 136
Kennedy et al. [47] (1987) the United States	RCT	Hospital	Discharge Planning is based on individual patient needs, emphasising communication with the patient and family	Care not described	Nurses	1 F/U	Control: 41 Exposed: 39
Kurant et al. [51] (2018) the United States	Not stated	Hospital	Laboratory-based utilisation management programs, including electronic health record (EHR) laboratory orders database	Usual service	Not applicable	8 months	160,000 EHR laboratory orders
Copeland et al. [28] (2017) the United States	NCBA	Hospital	Modelling of collective and individual oncologist per patient imaging counts	Before model		12 months	4605 patients
Pena et al. [58] (2014) the United States	NCBA	Hospital	Blood management program includes Improving communications and transfusion guidelines, Benchmarking using the issue-to-transfusion ratio and audits and gatekeeping of selected blood products	Before the Blood management program	The staff of the laboratory of the Blood Transfusion Service	36 months	All of the transfused components at MGH from 2010 to 2012
Weilburg et al. [80] (2017) the United States	Retrospective cohort	Hospital	Analysis of high-cost imaging utilisation in a stable cohort of patients cared for by PCPs during a 7-year period	Statewide high-cost imaging use data from a major private payer on the basis of the same claim set	Primary care physicians & speciality care physicians	84 months	109,823 patients
Konger et al .[50] (2016) the United States	NCBA	Hospital	Reductions in unnecessary clinical laboratory testing by using LES	pre-LES test volume	Pathologists	36 months	14,359 Exclusion Requests
El-Othmani et al. [33] (2019) the United States	Retrospective analyse	Hospital	The Joint Utilization Management Program	Before the Joint Utilization Management Program	Physicians, post-acute care providers, and inpatient interdisciplinary teams	12 before and 12 F/U	683 JUMP patient
Kim & Lee [48] (2020) Korea	Not stated	Medical Aid Beneficiaries	Case Management	Before Case Management	The case manager, a registered nurse or social worker,	12 Months	1741 case management clients
Wasfy et al. [79] (2019) the United States	Ret rospective cohort	Hospital	Hospital Readmissions Reduction Program	Pre-law trends	Not applicable	36 Months	3,038,740 total index hospital stays
Calsolaro et al. [25] (2019) Italy	Ret rospective analyse	Hospital	Potentially Preventable Readmission Grouping	Compering stand-alone admissions, index admissions and potentially preventable read missions	Geriatricians	30 days	1263 stand-alone admissions, 171 index admissions

Notes: *RCT* Randomised controlled trial, *ED* Emergency Department, *CM* Case Management, *NCBA* Non-controlled before-and-after studies, *LES* Laboratory expert system, *HIT* Health Information Technologies, *EMS* Emergency medical services, *POE* Physician’s order entry, *CDSS* Before Clinical Decision Support System, *HMO* Health maintenance organisation

Fourteen studies (21.9%) were randomized controlled trials [22, 23, 43, 47, 49, 52, 53, 55, 62, 66, 69, 70, 73, 74], three were multicenter research trials [36, 63, 76], two were quasi-experimental studies [31, 67], four were controlled before-and-after studies [30, 68, 72, 85], twenty-one studies (32.8%) were non-controlled before-and-after studies (NCBA) [24, 27–29, 32, 35, 37–39, 41, 42, 50, 54, 56–61, 75, 78], three were time-series studies [26, 34, 44], three were case-control studies [64, 65, 84], one was a prospective cohort study [77], one was longitudinal study, six were retrospective cohort studies [25, 33, 79–82], and four were cross-sectional studies [40, 45, 71, 83]. While, in two studies were not stated type of design [48, 51]. Fourty studies (59.7%) can be categorized as assessing interventions targeted at the patient journey during hospital stay or medical center-based interventions [22–24, 26, 27, 29, 30, 34, 37, 39, 40, 42, 44, 45, 49, 54, 56, 57, 59–63, 65, 69, 70, 72, 74, 75, 78, 81–83, 85]; four evaluated interventions aimed at discharge [41, 47, 55, 76], Not; and 13 examined community-based interventions [31, 35, 38, 43, 46, 52, 53, 64, 66–68, 73, 77].

### Methodological quality assessment

In the overall assessment, the methodological quality of only one reviewed study (1.5%) was rated as ‘strong’, while seven (11%) and 56 (87.5%) articles were rated as ‘moderate’ and ‘weak’, respectively (Appendix). In terms of study design, 21 studies (32.8%) were rated as ‘strong’. The remaining 13 studies (20.3%) scored ‘moderate’ and 30 studies (46.9%) scored ‘weak’. We were able to rate 39 studies for representativeness relating to withdrawals and drop-outs: 25 (64.1%) studies rated as ‘weak’, four (10.3%) as ‘moderate’, and ten (25.6) as ‘strong’. With respect to confounders, 11 (17.2%) studies were rated as ‘strong’, six (9.4%) as ‘moderate’, and 47 (73.4%) as ‘weak’. There were 23 studies (35.9%) rated as ‘weak’ for their data collection because the authors did not provide sufficient information on the validity or reliability of their collection methods. There were 37 papers (57.8%) rated as ‘moderate’ and four papers (6.3%) rated as ‘strong’. Based on the data analysis of each reviewed study, 36 (56.3%) of the reviewed studies were rated as ‘strong’, while 12 (18.8%) and 16 (25.0%) were rated as ‘moderate’ and ‘weak’, respectively. The reporting quality of the reviewed articles was also analysed. Out of the 64 articles included, 36 studies (56.3%) were rated as ‘strong’, 21 studies (32.8%) and seven studies (10.9%) were rated as ‘moderate’ and ‘weak’, respectively.

### Nine broad utilisation management methods

We identified nine broad utilisation management methods: care plan, case management, care coordination, utilisation review, clinical information system, physician profiling, consultation, education, and discharge planning. The findings related to these nine methods are described below in Table 2, using sub-categories of the following main types of interventions: non-organisational interventions aiming to reduce hospital utilisation, organisational interventions to reduce hospital utilisation, and interventions at the discharge stage of the patient journey.

**Table 2:** Reported measures and outcomes

Author (Year) Country	Type of intervention	Main Outcome Measure	Outcomes						Statistically significant (*P* < .05)
			Control			Intervention			
			Before	After	Difference	Before	After	Difference	
Sandberg et al. [66] (2015) Sweden	Case management	No. of admissions, mean	0.62	0.48		0.48	0.49		No
		LOS, mean	3.90	4.05		5.05	4.60		No
		No. of ED visits leading to hospitalization, mean	0.36	0.42		0.39	0.34		No
		No. of ED visits not leading to hospitalization, mean	0.22	0.37		0.15	0.08		Yes
		Proportion of ED visits not leading to hospitalisation	16 (38.1%)	23 (46.7%)		12 (27.9%)	4 (17.4%)		Yes
		No. of outpatient visits, mean	6.10	5.29		5.30	4.09		Yes
Haldiman et al. [40] (2014) the United States	Prospective review	No. of FFPs transfused per 1000 patients discharged per year	–	–	–	Y1: 66.7	Y4: 46.9	- 19.8 (−29.7%)	Yes
		No. of platelets transfused per1000 patient discharged per year	–	–	–	Y1: 23.7	Y2: 18.7	-5 (−21.1%)	Yes
		Annual cost savings	$130,000,000						NR
Goodnough et al. [37] (2014) the United States	Concurrent review	% of blood transfusions in patients whit HB levels exceeded 8 g/dl	–	–	–	57%	30%		Yes
		Total RBC transfusions	–		–	–	–	− 7186 (−24%)	NR
		Total plasma transfusions	–	–	–	–	–	−10%	NR
		Total platelets transfusions	–	–	–	–	–	−12%	NR
		All blood components	–	–	–	–	–	−19%	NR
		Net savings	$1,616,750						NR
Joo [46] (2014) the United States	Case management	No. of Admissions	–	–	–	Y1: 0.62	Y2: 0.47		Yes
		Total LOS	–	–	–	Y1: 3.05	Y2: 2.28		No
		NO. of ED visits	–	–	–	Y1: 0.38	Y2: 0.36		No
		Symptom control	–	–	–	B: 4.07 Y1: 4.19	Y2: 4.27		Yes
		Quality of life	–	–	–	B: 3.89 Y1: 4.01	Y2: 4.03		Yes
		Personal well-being	–	–	–	B: 4.09 Y1: 4.13	Y2: 4.14		No
Buckley et al. [24] (2013) the United States	Drug-utilisation management program	The proportion of patients prescribed epoetin	–	–	–	2.4%	1.6%		Yes
		No. of patients inappropriately prescribed epoetin	–	–	–	184/496 (37.1%)	37/300 (12.3%)		Yes
		Total no. of epoetin units administered	–			24,531,340	13,511,800	−45%	Yes
		Total epoetin costs	–	–	–	$220,786 ($36,797/mo)	$121,606 ($20,268/mo)	−45%	Yes
		% of total costs was attributed to inappropriate epoetin prescribing	–	–	–	36.8%	13%		Yes
		Annual cost savings	$ 198,352 ($ 16,529/mo)						Yes
Reinius et al. [62] (2013) Sweden	Case management	No. of ED visits	–	6.4	–	–	4.9	–	RRs 0.77; 95% CI 0.69-0.87
		No. of admissions, mean	–	2.1	–	–	1.7	–	No
		No. of hospital days per patient per year	–	16.9	–	–	7.0	−58%	Yes
		No. of out-patient visits, mean	–	25.4	–	–	21.4	−15.7%	RRs 0.85; 95% CI 0.79–0.90
		Costs per patient per year	–	€26,490	–	–	€11,417	−57%	Yes
		Quality-of-life scores	–	–	–	–	–	–	Yes
Crane et al. [30] (2012) the United States	Case management	No. of ED visits, median	6.96	5.04	−1.92	6.96	2.76	−4.2	Yes
		Total ED and inpatient charges per patient per mon, mean	–	–	–	$1167	$230	-$937	Yes
Roland et al. [64] (2012) the United Kingdom	Case management	No. of emergency admissions	–	–	–	–	–	+ 9%	Yes
		No. of elective admissions	–	–	–	–	–	−21%	Yes
		No. of out-patient visits	–	–	–	–	–	−22%	Yes
		Inpatient and out-patient costs	–	–	–	–	–	-£223 −9%	Yes
Koehler et al. [49] (2009) the United States	Care coordination	No. of 0-30 day post-discharge readmissions/ ED visits	–	8 (38%)	–	–	2 (10%)		Yes
		No. of 31-60 day post-discharge readmissions/ED visits	–	1 (4.8%)	–	–	4 (20%)		No
		Total post-discharge readmissions/ED visits at 60 days	–	9 (42.9%)	–	–	6 (30%)		No
Schraeder et al. [67] (2008) the United States	Case management	Admissions, %	–	53.8	–	–	51	–	No
		Hospital bed days, mean	–	13.89	–	–	8.19	–	Yes
		ED visits, mean	–	1.79	–	–	1.48	–	No
		Readmissions	–	28.8%	–	–	19.2%	−34%	Yes
		Cost of care per patient per mon, mean	–	$708	–	–	$1193	-$485	Yes
		Adjusted cost of care per patient per mon (cost savings)	–	–	–	–	–	$106	No
Holsinger et al. [42] (2008) the United States	Collaborative model	1-day hospital stays	–	–	–	–	–	−19%	NR
Sweeney et al. [77] (2007) the United States	Patient-centred management	No. of admission, mean	–	1.9	–	–	1.2	−36.8%	Yes
		Hospital days, mean	–	13.4	–	–	8.5	−36.6%	Yes
		No. of ED visits, mean	–	1.5	–	–	1.0	−33.3%	No
		Rehabilitation days, mean	–	5.8	–	–	3.7	−36.2%	No
		Hospice days, mean	–	2.4	–	–	3.3	37.5%	No
		Home care days, mean	–	30.9	–	–	36.8	26.6%	No
		The overall cost per patient for 18 mon, mean	–	$ 68,341	–	–	$ 49,742	$ -18,599 (−27.2%)	NR
Phillips et al. [59] (2006) Australia	Case management	Admissions, sum of the percentage	–	–	–	1104	931		No
		No. of ED visits, mean	–	–	–	10.2	13.0	+ 2.8 (27.4%)	No *P* = 0.55
		ED LOS, minutes, mean	–	–	–	297	300	+ 3	No
		No. of ED overnight observation, mean	–	–	–	1.3	3.4	+ 2.1 (166%)	Yes
		Housing stability score	–	–	–	3.6	4.1	0.5 (14%)	Yes
		Primary care engagement score	–	–	–	2.6	3.1	0.5 (19%)	Yes
		Community care engagement score	–	–	–	2.1	3.2	1.1 (52%)	Yes
		Drug and alcohol use	–	–	–	68.3%	58.9%		No
Sledge et al. [73] (2006) the United States	Case management	No. of admissions, mean	2.0	1.7	−0.3	1.9	1.3	−0.6	No
		No. of ED visits, mean	3.3	2.7	−0.6	2.0	1.5	−0.5	No
		No. of clinic visits, mean	5.9	5.7	−0.2	6.4	7.9	+ 1.5	Yes
		Total cost, mean	$17,721	$15,447	-$2274	$17,265	$16,291	-$974	No
		SF-36 Mental Health Function Score	21.7	22	0.3	21.3	21.4	0.1	No
		Overall patient satisfaction	7.24	6.7	−0.54	7.47	7.6	0.13	No
Mahendran et al. [54] (2006) Singapore	Case management	No. of readmissions	–	–	–	65	26	−39	Yes
		No. of patients who defaulted follow-up appointments	–	–	–	All outpatient: 24%	CM patient: 11.9%		Yes
		No. of days per admission, mean	–	–	–	15.6	4	−11.6	Yes
Zemencuk et al. [85] (2006) the United States	Physician profiling	LOS	–	–	–	–	–	− 0.32 day	Yes
Latour et al. [52] (2006) the Netherlands	Case management	Readmission rate	–	11 (15.9%)	–	–	16 (20.6%)	–	No
		Quality of life	–	–	–	–	–	–	No
		Psychological functioning	–	–	–	–	–	–	No
Hegney et al. [41] (2006) Australia	Discharge planning using risk screening tool	ED revisitation rate	–	–	–	21%	5%	−16%	Yes
		Readmission rate	–	–	–	9 (10.2%)	7 (4.7%)	−2 (5.5%)	No
		ALOS	–	–	–	6.17	5.37	−0.8	NR
Horwitz et al. [43] (2005) the United States	Case management	No. of admission	–	7/109 (6.4%)	–	–	3/121 (2.5%)		No
		No. of ED visits	–	32/109 (29.4%)	–	–	38/121 (31.4%)		No
		Primary care contact in 60 days	–	15/109 (13.8%)	–	–	62/121 (51.2%)		Yes
		Cost of an ED visit, mean	$330	$319		$330	$243		NR
Leung et al. [53] (2004) China	Case management	Total no. of admissions, mean	1.4	2.7		3.0	2.3		Yes
		Total no. of hospital bed days, mean	6.8	10.7		12.9	9.6		Yes
		Total no. of visits, mean	0.4	0.8		0.5	0.3		No
		Total no. of outpatient visits, mean	6.7	6.9		9.0	8.3		Yes
Cox et al. (2003) [29] the United States	Case management	No. of admissions, mean	–	–	–	3.11	0.82	−2.29	Yes
		Hospital days, mean	–	–	–	46.6	12.4	−34.2	Yes
		Cost-saving per inpatient day	–	–	–	–	–	$ 166	Yes
Hwang et al. [44] (2002) Korea	Physician’s order entry system	LOS, mean	–	–	–	11.4	8.2	−3.2	Yes
		No. of daily orders	–	–	–	10.9	18.9	+ 8	Yes
		No. of stat lab tests	–	–	–	3.3	1.8	−1.5	Yes
Fateha [34] (2002) Bahrain	Concurrent Review	LOS, mean	–	–	–	8.3	6.6	−1.7 (−20.5%)	Yes
Ferrazzi et al. [35] (2001) Canada	Advanced life support drug treatment given by ambulance attendants	Proportion of admissions	–	–	–	145 (67.4%)	102 (54.3%)		Yes
		ED LOS, min, mean	–	–	-	206.9	220.9	−14	No
		Ambulance scene time, min	–	–	–	12.3	14.2		Yes
Okin et al. [57] (2000) the United States	Case management	No. of ED visits, median	–	–	–	15	9	−6 (−40%)	Yes
		No. of out-patient visits, median	–	–	–	2	4		Yes
		No. of admissions, median	–	–	–	1	1		No
		Medical inpatient days, median	–	–	–	5	2		No
		ED costs, median	–	–	–	$4124	$2195	$-1938	Yes
		Medical inpatient costs, median	–	–	–	$8330	$2786	$-1082	Yes
		Medical out-patient costs, median	–	–	–	$476	$612	$94	No
		Homelessness	–	–	–	35	15	−20 (−57%)	Yes
		Alcohol use	–	–	–	37	29	−8 (−22%)	Yes
		Drug use	–	–	–	27	20	−7 (−26%)	Yes
		Linkage to primary care	–	–	–	–	–	+ 74%	Yes
		Net cost savings	$132,726						NR
Bates et al. [22] (1999) the United States	Computerised physician order entry	No. of clinical laboratory orders that were cancelled in response to reminders	–	Not applicable	–	–	300 of 437 (69%)	–	Yes
		The proportion of the redundant tests that were performed	–	257 (51%)	–	–	117 (27%)	–	Yes
		Annual lab cost savings	$35,000						NR
Wickizer et al. [82] (1998) the United States	Utilisation management strategies	No. of days approved	–	–	–	–	–	−50%	Yes
Spillane et al. [74] (1997) the United States	Case management	No. of ED visits, median	13	6	−7	14	7	−7	NO
Bree et al. [23] (1996) the United States	Pre-certification	No. of examinations per admission, mean	–	4.4	–	–	4.4	–	No
		LOS, mean	–	6.1	–	–	6.0	–	No
		% of patients with one or more tests	–	88.7%	–	–	88%	–	No
		Relative value units (RVUs), mean.	–	336.0	–	–	356.1	–	No
		Adjusted RVUs	–	−10.2	–	–	−8.8	–	No
Shea et al. [69] (1995) the United States	Clinical information system	Adjusted LOS, mean	–	0.012	–	–	−0.011	−2.3%	Yes
Cardiff et al. [26] (1995) Canada	Utilisation management	Inappropriate admissions	C: 26 (18%) D: 36 (23%)	C: 18 (13%) D: 48 (30%)	–	A: 71 (24%) B: 78 (26%)	A: 88 (29%) B: 68 (23%)	–	Among hospitals in both time period: Yes
		Adjusted inappropriate continued days of stay	C: 0.0656 D: 0.0617	C: 0.0665 D: 0.0906	–	A: 0.1597 B: 0.1224	A: 0.0770 B: 0.0918	–	B: Yes A,C,D: No
		30-day readmission (rate per 1000 discharge)	C: 105 D: 92	C: 96 D: 76	–	A: 83 B: 73	A: 71 B: 60	–	A,B,D: Yes C:No
Styrborn [76] (1995) Sweden	Discharge planning	Adjusted LOS	–	B: 10.5 C: 10.9	–	–	A: 9.6	A-(B + C): −1.1	No
		No. of bed-blocking patients	–	B: 35 C: 35	–	–	A: 31	−4	NR
		Waiting days/patient	–	B: 11.3 C: 18.0	–	–	A: 8.2	A-(B + C): −6.4	Yes
		Charge days per patient	–	B: 6.2 C: 13.4	–	–	A: 4.2	A-(B + C): −5.6	Yes
Rosenberg et al. [65] (1995) the United States	Utilisation review, second opinion, discharge planning, case management	No. of out-patient procedure	–	913	–	–	789	−124	Yes
		No. of inpatient procedure	–	452	–	–	466	14	No
		No. of admission per 1000 patients		625.4			641.8	16.4	No
		Adjusted LOS	–	5.9	–	–	6.1	0.2	No
		Adjusted ALOS, mean	–	5.8	–	–	6.1	0.3	No
Jambunathan et al. [45] (1995) the United States	Case management	No. of case management visits/Adjusted LOS (r-value)	–	–	–	–	.6138	–	Yes
Williams et al. [83] (1994) Australia	Drug utilisation review	No. of patients using benzodiazepines	–	–	–	30 (40%)	15 (20%)	−15 (−20%)	Yes
		No. of patients using potentially adverse side-effects drug combinations (%)	–	–	–	21 (28%)	7 (9.3%)	−14 (− 18.7%)	Yes
Wickizer [81] (1992) the United States	Utilisation review	No. of admissions	–	–	–	–	–	−12%	Yes
		Adjusted LOS	–	–	–	–	–	–	No
		Hospital routine costs	–	–	–	–	–	−8%	Yes
		Hospital ancillary costs	–	–	–	–	–	−9%	Yes
		Total medical cost	–	–	–	–	–	− 6%	Yes
		Cost savings per employee per year	$115						NR
Woodside et al. [84] (1991) the United States	Utilisation management strategies	Adjusted LOS	–	11.8	–	–	9.1	−23%	NR
		Total costs, mean	–	$22,695	–	–	$19,042	−16%	NR
Silver et al. [71] (1992) the United States	Prospective review	No. of orders cancelled	–	–	–	–	114 (21%)	–	NR
		Medical costs	–	–	–	–	–	-$22,000	NR
Fowkes et al. [36] (1986) the United Kingdom	Utilisation review	No. of X-ray tests per100 operations	–	–	–	29.4	13.3	−16.1	Yes
Echols et al. [32] (1984) the United States	Drug utilisation review	No. of antibiotic treatment courses	–	–	–	–	–	−30%	Yes
		No. of patients receiving any antibiotic	–	–	–	47%	30%	−17%	Yes
Restuccia [63] (1982) the United States	Utilisation review	No. of inappropriate days, mean	–	D: 3.25	–	–	A: 2.59 B: 2.75 C: 3.25	A-D: −0.66 B-D: −0.5 C-D: 0	Yes
		Adjusted LOS, mean	–	D: 14.59	–	–	A: 12.23 B: 13.81 C: 15.23	A-D: −2.36 B-D: −0.78 C-D: 0.64	Yes
Murphy [56] (2014) the United States	Case management	No. of ED visits	–	–	–	7	2	−5	Yes
		No. of out-patient visits	–	–	–	7	2	−5	Yes
		Direct treatment costs	–	–	–	$2328	$1043	-$1285	Yes
		Direct treatment cost per visit	–	–	–	$323	$235	-$88	Yes
		Net income	–	–	–	-$608	-$177	$431	Yes
Chiang et al. [27] (2014) Taiwan	Case management	No. of ED visits, mean	–	–	–	63	26	−37 (−58%)	Yes
Pillow et al. [60] (2013) the United States	Care plans	No. of ED visits per year per patient	–	–	–	22.6	21.2	−1.4	Yes
		No. of admissions per year per patient	–	–	–	7.3	6.8	−0.5	No
Dehaven et al. [31] (2012) the United States	Community-based partnership	No. of ED visits, mean	–	1.44	–	–	0.93	–	Yes
		No. of hospital days, mean	–	1.07	–	–	0.37	–	Yes
		Direct hospital costs, mean	–	$1188	–	–	$445.6	−62%	Yes
		Indirect costs, mean	–	$692.1	–	–	$313.3	−55%	Yes
Tadros et al. [78] (2012) the United States	Case management	No. of EMS visits, median	–	–	–	8	4	−4	Yes
		Total no. of EMS visits	–	–	–	736	459	−37*.*6%	Yes
		No. of ED visits, median	–	–	–	1	0	−1	No
		Total no. of ED visits	–	–	–	199	143	−28.1%	No
		No. of admissions, median	–	–	–	0	0	0	No
		Total no. of admissions	–	–	–	33	30	−9.1%	No
		LOS, median	–	–	–	0	0	0	No
		LOS, days	–	–	–	122	88	−27.9%	No
		EMS costs	–	–	–	$689,743	$468,394	−32.1%	Yes
		Out-patient costs	–	–	–	$413,410	$360,779	−12*.*7	No
		Inpatient costs	–	–	–	$687,306	$646,881	−5.9%	No
		Total costs	–	–	–	$1,790,459	$1,476,053	-$314,406 (−17.6%)	NR
Shah et al. [68] (2011) the United States	Care management	No. of ED visits per year, median	–	–	–	6.0	1.7	−3.9	Yes
		No. of admissions, median	–	–	–	0.0	0.0	0.0	No
		Unadjusted ED cost per patient per year, mean	–	–	–	$2545	$1874	-$671 (−26%)	Yes
		Unadjusted admission cost per patient per year, mean	–	–	–	$ 20,298	$ 7053	-$ 13,245 (−65%)	Yes
Stokes-Buzzelli S et al. [75] (2010) the United States	Health Information Technologies	No. of ED visits, mean	–	–	–	67.4	50.5	−16.9 (−%25)	Yes
		ED LOS, min	–	–	–	388	342	−46 (−%12)	No
		Lab studies ordered, mean	–	–	–	1847	1328	−519 (−%28)	Yes
		ED charges	–	–	–	$64,721	$49,208	−15,513 (−24%)	Yes
		Total Emergency Department Contact Time, hours	–	–	–	443.7	270.6	− 173.1 or 7.21 days (−39%)	Yes
Grimmer- Somers et al. [38] (2010) Australia	Individualised care plan	No. of ED visits	–	–	–	0.81	0.59		NR
		No. of admissions	–	–	–	0.32	0.21		NR
		LOS	–	–	–	–	–	−1.3	NR
Grover et al. [39] (2010) the United States	Case management	No. of ED visits, mean	–	–	–	13.8	3.6	−74%	Yes
		No. of CT images	–	–	–	153.6	61.2	−60%	Yes
Skinner et al. [72] (2009) the United Kingdom	Case management	No. of ED visits, median	–	–	–	12	6	−6	Yes
		Total no. of ED visits	–	–	–	720	499	− 221 (−31%)	Yes
Shumway et al. [70] (2008) the United States	Case management	No. of ED visits, mean	5.2	2.0		3.6	0.9		Yes
		No. of admissions, mean	0.9	0.3		0.8	0.3		No
		Medical inpatient days, mean	3.4	1.7		3.4	1.3		No
		No. of outpatient visits, mean	2.5	2.6		2.7	2.2		No
		ED costs, mean	942	647		790	247		Yes
		All hospital costs, mean	8423	3849		8508	4761		No
		Homeless, n (%)	32 (80)	11 (33)		61 (76)	22 (32)		Yes
		Problem alcohol use, n (%)	21 (53)	12 (30)		38 (48)	22 (28)		Yes
		No. of health insurance (%)	31 (78)	17 (53)		59 (75)	30 (44)		Yes
		No. of social security income (%)	29 (74)	18 (58)		63 (79)	26 (43)		Yes
		Basic financial needs, mean	4.4	3.7		5.2	3.8		Yes
		Psychiatric symptoms (total BSI score), mean	10.0	9.8		11.6	10.4		No
Pope et al. [61] (2000) Canada	Case management	No. of number of ED visits, median	–	–	–	26.5	6.5	−20	Yes
		Total no. of ED visits	–	–	–	616	175	− 441 (−72%)	Yes
Moher et al. [55] (1992) Canada	Discharge planning	LOS, mean	–	9.4	–	–	7.43	−1.97	Yes
		Readmission rate at 2 weeks	–	18 (14%)	–	–	22 (16%)		No
Kennedy et al. [47] (1987) the United States	Discharge planning	LOS, mean	–	9.7	–	–	7.8	−1.9	Yes
		Readmission rate at 8 weeks	–	14 (34%)	–	–	11 (28%)	−6%	NR
Kurant et al .[51] (2018) the United States	Laboratory-based utilisation management programs								
Copeland et al. [28] (2017) the United States	Modelling	Total imaging per patient	–	–	–	–	–	–	RRs 1.93; 95% CI 1.67–2.23
Pena et al .[58] (2014) the United States	Blood management program, benchmarking	Total RBC transfusions	–	–	–	37,167	34,602		Yes
		Total plasma transfusions	–	–	–	–	10,544		NR
		Total platelets transfusions	–	–	–	8202	7844		NR
		Total albumin transfusions	–	–	–	23,949	24,557		NR
		Total IVIg transfusions	–	–	–	52,085	44,973		
Weilburg et al. [80] (2017) the United States	Analysis of high-cost imaging utilisation	No. of high-cost imaging per year	–	–	–	0.43 examinations	0.34 examinations	- 21.3%	Yes
		Overall laboratory utilisation	–	–	–	–	–	−9.4%	Yes
		Inpatient stays	–	–	–	0.453	0.422		No
		No. of departments visited	–	–	–	0.558	0.823		Yes
Konger et al. [50] (2016) the United States	Reductions in unnecessary clinical laboratory testing	Total test volume per year	–	–	–	–	–	−11.18%	Yes
El-Othmani et al. [33] (2019) the United States	Joint utilisation management program	LOS	9.27	6.2		4.22	3.04		
		The rate of 30 day readmission	21.05	23.50		9.94	8.0		
		Inpatient rehabilitation	15.79	5.88		5.9	3.08		
Kim & Lee [48] (2020) Korea	Case Management	Inpatient days	30.5	10.6					
		Outpatient visits	128.3	104.7					
		Self-care ability	15.41	18.64					
Wasfy et al. [79] (2019) the United States	Hospital Readmissions reduction Program	In-patient readmission	0.023	0.002					yes
		Treat-and-discharge visit to emergency department	0.014	0.029					yes
		Observation stay (not leading to inpatient readmission)	0.019	0.024					yes
Calsolaro et al. [25] (2019)	Hospital Readmissions Reduction Program	Potentially preventable read-missions (PPR)							
		LOS (median and range)	5 (4-6)			6 (2-14)			

## Prehospital advanced life support drug treatment

These interventions focused on access to primary care, medical and social resources. For example, two studies [31, 68] evaluated interventions that aimed to improve access to primary care. Studies suggest that improving access to primary care centres is associated with fewer ED visits [31, 68], fewer inpatient hospital days than controls [31], but report no difference in inpatient admissions between groups [68]. One retrospective cohort study examined the effect of prehospital advanced life support drug treatment in reducing subsequent hospital utilisation by the medical patients receiving such drugs [35]. There was a significant decrease in admissions in the drug intervention group driven by chest pain patients and improved prehospital field conditions for all chief complaints. Care plan and case management were the main interventions related to prehospital advanced life support drug treatment.

Two comparative cohort studies examined the impact of patient care plans on service utilisation [38, 77]. Sweeney et al. [77] compared patient-centred management to usual case management for patients who had a life-limiting diagnosis with multiple comorbid conditions. Among the patient-centered management, inpatient admissions reduced by 38%, inpatient hospital days by 36%, and emergency department visits by 30%. Grimmer-Somers et al. [38] found that a holistic community-based program using a care plan for frequent ED attendees had significant improvements in client health and decreased crisis emergency department and inpatient admissions.

## Case management

### Primary care case management

Case management is “a collaborative process that assesses, plans, implements, coordinates, monitors, and evaluates the options and services required to meet an individual’s health needs using communication and available resources to promote quality and cost-effective outcomes” [50]. Eight studies focused on using case management interventions based outside the hospital. Five studies reported a decrease in hospital utilisation [45, 46, 64, 66]. Three studies found no significant difference between groups in neither ED visits nor hospital admissions [43, 67, 73].

### Hospital-based case management

Of 23 studies evaluating case management interventions, 12 focused on case management as an ED-initiated or medical centre-based intervention for frequent hospital utilisers. Six comparative cohort studies observed a decrease in the mean or the median number of ED visits than the controls [30, 72] or before the case management [27, 39, 57, 61]. One study reported an increase of 2.79 median ED visits post-intervention [59]. This study included primarily patients with substance abuse or psychiatric problems underlying the ED visits, suggesting case management may be less effective in reducing ED utilisation in this population. One RCT reported no significant difference in the median number of ED visits following CM [74]. In contrast, two RCTs reported a decrease in the number of ED visits [62, 70] and hospital days [64] among those in the intervention group. Two studies have examined changes in hospital admissions or LOS, found a significant decrease in the number of admissions [29], hospital readmissions [54] and LOS.

## Care coordination

Two studies examined the impact of care coordination programs on ED visit rate amongst frequent ED users [49, 56]. The randomised controlled pilot study by Koehler et al .[49] found that hospital-based care coordination using extra care bundle comprising three interventions (medication counselling, enhanced discharge planning, and phone follow-up) targeting high-risk older people compared to usual care was successful in reducing 30-day post-discharge hospital readmission or emergency department visit rates. The comparative cohort study by Murphy et al. [56] implemented a multidiscipline ED-care coordination program using a regional hospital information system capable of sharing patients’ individualised care plans between ED providers. The study reported a significant decrease in ED visits 12-months following the intervention.

## Utilisation Review

The utilisation review program consists of several different review activities: pre-admission authorisation (prospective review), concurrent review (during the patient stay), retrospective review (relying on medical records), prospective review. One study investigating a pre-admission review program found a decrease in hospital admissions by approximately 12% [81]. Of eight studies that examined the effect of concurrent review on the LOS, five studies found a decrease in hospital LOS [26, 34, 63, 82, 84]. Another study that examined the effect of utilisation review on patterns of health care use found that the referrals for a second opinion have reduced the number of procedures performed in the review group. However, there was no significant difference between the groups during the study period in terms of rates of admission to medical-surgical, substance abuse, or psychiatric units, average LOS, the percentage of those who received pre-admission testing, or the rates of use of home care following utilisation review activities [65].

A retrospective analysis of utilisation management programs has concluded that pre-admission review rarely denies requests for admission, and nearly one-third of patients approved by pre-admission review for inpatient care requested approval for continued stay through concurrent review [82]. One multicenter trial examined the effect of utilisation management strategies on the use of a radiological test [36]. There was a consistent reduction from 29.4 to 13.3 X-rays per 100 operations after introducing the new request form and concurrent review. Two studies that evaluated the effectiveness of a prospective review program in reducing blood component utilisation reported that the implementation by the blood bank staff of a prospective review of orders for blood products resulted in a significant decrease of 38.8% and 31.4% in the use of fresh frozen plasma and platelets, respectively [40], as well as a total reduction inpatient medical costs realised as a result of cancelled orders [71]. Due to the importance of drug utilisation, this type of utilisation review has been categorised as a primary intervention.

### Drug utilisation review

Three studies focused on drug utilisation review interventions. One study reported a significant decrease in the number of antibiotic treatment courses and the percentage of patients receiving any antibiotic following implementing an antibiotic order form for all inpatient antibiotic orders in the hospital [32]. The second study reported a significant decrease from 40% to 20% of patients using benzodiazepines after drug utilisation review activities in an inpatient setting [83]. Another retrospective cohort study examined the effect of implementing a drug utilisation management program and evidence-based guidelines on the appropriate use of drugs and found that implementing a drug-utilisation management program using clinical pharmacists was associated with a decrease in inappropriate epoetin prescribing and significant cost savings [24].

## Clinical information system

A clinical information system is a computer-based system encompassing clinical or health-related information, distinguished from administrative information systems by the requirement for data entry or data retrieval by clinicians at the point of care. Some areas addressed by clinical information systems are clinical decision support, electronic medical records, physician’s order entry, telemedicine, problem lists, summary reports, results review, nursing protocols and care plans, and alerts and reminders. Recently, interests have been focusing on medical errors with monitoring and managing variation in practice [86]. Electronic medical records and physician’s order entry systems, and clinical decision support are the primary interventions related to clinical information systems.

### Electronic Medical Record

One before-after analysis of an intervention targeting ED frequent users reported that the use of health information technologies to identify the most frequently visiting patients and easy access to individualised care plans through the EMR to all healthcare providers resulted in a significant reduction in the number of ED visits, labs ordered, total ED contact time, and ED charges [75].

### Physician’s order entry system

A physician’s order entry system is a subsystem of a hospital information system. One prospective time series study reported that the number of stat lab tests and overall LOS at six months after physician’s order entry implementation decreased significantly compared with the pre- physician’s order entry system period [44]. Using a randomised controlled design, Shea et al. [69] demonstrated that a computer-generated informational message directed to physicians as an intervention resulted in reduced LOS in an inpatient setting. According to Bates et al. [22], 69% of potentially redundant diagnostic tests were cancelled in response to reminders following the introduction of a clinical information system that included a physician’s order entry system.

### Clinical decision support

A clinical decision support system is a computer-based application that analyses data and provides knowledge and person-specific information to aid physicians and other health providers in clinical decision making [87]. One study that evaluated real-time clinical decision support intervention observed improved blood utilisation. After implementing clinical decision support system, the percentage of patients transfused outside the guidelines decreased to 35% [37].

## Physician profiling

Physician profiling is a cost-containment strategy whereby the patterns of health care provided by a practitioner or other provider (e.g., hospital) for the defined population are compared to other norms - profiles of other physicians or practice guidelines - based on practice [88]. A quasi-experimental study with control groups found that LOS at the profiled site decreased by an additional third of a day in the profiling year than at the non-profiled sites [85].

## Consultation

The randomised controlled trials by Bree et al. [24] implemented mandatory radiology consultation whereby each radiology examination required prior approval. This intervention did not observe differences in inpatient imaging use following the mandatory radiology consultation.

## Discharge planning

Discharge planning refers to developing a plan to treat the patient’s medical needs after leaving the inpatient department to contain costs and improve patient outcomes. Discharge planning should ensure that patients leave the hospital at an appropriate time in their care and that, with adequate notice, the provision of post-discharge services is organised [89]. We identified three studies that focused on interventions at the discharge stage of the patient journey [41, 47, 55]. All three studies that examined the effect of discharge planning on LOS in hospital and readmission rates compared with usual care found a decrease in hospital LOS for those allocated to discharge planning. There were lower readmission rates in the discharge planning group for older participants with a medical condition at three months of discharge [41, 47].

### Early supported discharge

Discharge planning typically involves a greater degree of care provision and support following discharge than discharge planning interventions. Early supported discharge or early home-supported discharge may include discharge planning but aims specifically to accelerate discharge from the hospital with continued support in a community setting, typically at the same intensity that would have been provided had the patient remained in hospital. These interventions are usually provided by multidisciplinary teams, including doctors, nurses, and therapists. Still, the degree of coordination and whether they are driven by hospital outreach or community teams can vary [89].

### Post-discharge case management

Two RCTs have examined the effectiveness of case management provided after patients are discharged from the hospital regarding the utilisation of hospital services by these patients. One study found a significant reduction in hospital admissions, bed-days and attendances at the out-patient department [53]. In contrast, the second study did not find significant differences between groups for readmission, care utilisation, quality of life, or psychological functioning [52].

### Cost outcome

Of all included studies, 23 studies provided cost-related outcomes. Six studies reported savings after implementing utilisation review programs [24, 37, 40, 81, 84] or a computerised physician order entry system [22]. One study reported cost savings from reduced days of hospitalization [29]. Ten studies reported significantly reduced hospital charges [30, 31, 56, 62, 64, 67, 68, 77] or ED costs after the intervention [43, 75]. One randomised controlled trial of 96 patients observed a trend toward reduced total healthcare cost in the experimental group, but the difference was not statistically significant [73]. Two studies reported a mixed effect - one reported a significant decrease in ED and medical inpatient costs but no apparent change in the cost of medical out-patient, psychiatric inpatient, psychiatric emergency, or ambulance services [57]. The other found a significant decrease in ED costs. However, no difference was reported for inpatient services, psychiatric emergency services, out-patient services, physicians’ fees, or total hospital costs, with the cost of case management included [70]. Also, one study reported program costs with no assessment of net costs or savings [38].

## Education

Developing education programs for patients, families and health care providers (i.e., nurses or physicians) is considered the primary intervention in many countries [49, 67, 77, 90]. The goal of the education programs is to provide health care providers with the principles of utilisation management.

## Discussion

Our review identified nine utilisation management methods, including care plan, case management, care coordination, utilisation review, clinical information system, physician profiling, consultation, education, and discharge planning. Of all interventions reported in the reviewed studies, case management strategy was the most frequently examined. Disease management is considered an effective strategy for dealing with frequent hospital users with specific diseases (e.g., congestive heart failure or diabetes). Whereas disease management focuses on particular illnesses, case management is focused on optimising multidisciplinary treatment. We identified several models of case management, such as brokerage [54], assertive community treatment [46], intensive case management [29, 39], clinical case management [57, 70], and different case management models (i.e., strengths-based case management, generalist case management, rehabilitation).

Our findings suggest that interventions aimed to increase primary care accessibility and case management effectively reduce ED visitation [31]. Though mostly uneven in methodological rigour, studies indicate that pre-admission review for hospitalisation is highly effective in reducing hospital admissions. The implementation of utilisation management interventions increased out-patient visits, possibly reflecting the link of frequent hospital users to other services. Overall, studies that focused on interventions during the patient stay in the hospital (e.g., concurrent review) and interventions at the discharge stage of the patient journey (e.g., discharge planning) effectively reduce the LOS. However, the limited evidence showed that mandatory radiology consultation interventions were ineffective in reducing inpatient imaging use. As a good outcome, introducing the clinical information systems (e.g., physician’s order entry system) reduced LOS. Such automated access to patient records improved the efficiency of information exchange among physicians across the continuum of care. Clinical decision support systems, which consisted of interruptive best practice alerts at the physician’s order entry system, also significantly improved blood utilisation. We found that interventions directed towards supply, such as physician profiling, were associated with decreased LOS without adversely affecting physician satisfaction. However, such reductions were also observed among control groups in ED visit numbers [30, 70, 73, 74], hospital admissions [66, 70, 73] and LOS [70]. Case or care management and utilisation review interventions were consistently reported to reduce hospital costs, and no studies reported increases in hospital costs following the intervention.

There were several limitations to this review. First, there is marked heterogeneity among reviewed studies. Second, in an attempt to focus on the literature concerning the general adult frequent user populations, studies were excluded that did not examine a general population (e.g., pediatric, individuals with asthma, cancer, diabetes, and cardiovascular disease) or focused on a specialised out-patient care setting.

## Conclusion

To ensure the delivery of efficient and effective health care, to reduce the misuse of inpatient and outpatient services, the use of utilisation management strategies in hospitals is unavoidable. The use of relevant strategies and interventions allows for avoiding unintended consequences emanating from the financial incentives and disincentives on health care professionals’ decisions around care and service delivery.

## Data Availability

The data are openly available upon request from the corresponding author.

## Appendix

**Appendix Table:** Quality assessment of included studies

Authors (year)	Study Design	Blinding	Selection Bias	Withdrawals/ Drop-Outs	Confounders	Data Collection	Data Analysis	Reporting	Overall
1. Sandberg et al. (2015) [66]	Strong	Weak	Strong	Strong	Weak	Strong	Strong	Strong	Strong
2. Haldiman et al. (2014) [40]	Moderate	No rating	Weak	No rating	Weak	Weak	Weak	Weak	Weak
3. Goodnough et al. (2014) [37]	Weak	No rating	No rating	No rating	Weak	Moderate	Moderate	Weak	Weak
4. Joo (2014) [46]	Moderate	No rating	Weak	Weak	Weak	Strong	Strong	Strong	Weak
5. Buckley et al. (2013) [24]	Weak	No rating	No rating	No rating	Weak	Moderate	Weak	Strong	Weak
6. Reinius et al. (2013) [62]	Strong	Moderate	Moderate	Strong	Weak	Weak	Strong	Strong	Weak
7. Crane et al. (2012) [30]	Strong	Weak	Weak	Weak	Weak	Weak	Moderate	Strong	Weak
8. Roland et al. (2012) [64]	Moderate	No rating	Weak	Moderate	Weak	Weak	Moderate	Moderate	Weak
9. Koehler et al. (2009) [49]	Strong	Weak	No rating	Strong	Weak	Moderate	Strong	Strong	Weak
10. Schraeder et al. (2008) [67]	Weak	No rating	Weak	Weak	Strong	Weak	Strong	Strong	Weak
11. Holsinger et al. (2008) [42]	Weak	No rating	Weak	Weak	Weak	Weak	Weak	Weak	Weak
12. Sweeney et al. (2007) [77]	Strong	No rating	Weak	Strong	Weak	Moderate	Weak	Strong	Weak
13. Phillips et al. (2006) [59]	Weak	No rating	Weak	Weak	Weak	Moderate	Strong	Strong	Weak
14. Sledge et al. (2006) [73]	Strong	Moderate	Moderate	Strong	Weak	Weak	Strong	Strong	Weak
15. Mahendran et al. (2006) [54]	Weak	No rating	Weak	Weak	Weak	Weak	Weak	Moderate	Weak
16. Zemencuk et al. (2006) [85]	Strong	Weak	Weak	Weak	Strong	Weak	Strong	Strong	Weak
17. Latour et al. (2006) [52]	Strong	Weak	Moderate	Strong	Weak	Weak	Strong	Strong	Weak
18. Hegney et al. (2006) [41]	Weak	No rating	Weak	Weak	Weak	Moderate	Strong	Moderate	Weak
19. Horwitz et al. (2005) [43]	Strong	Weak	Weak	Weak	Weak	Weak	Strong	Moderate	Weak
20. Leung et al. (2004) [53]	Strong	Weak	Weak	Weak	Weak	Moderate	Strong	Strong	Weak
21. Cox et al. (2003) [29]	Weak	No rating	Weak	Weak	Weak	Weak	Weak	Moderate	Weak
22. Hwang et al. (2002) [44]	Moderate	No rating	Strong	Weak	Weak	Moderate	Strong	Strong	Weak
23. Fateha (2002) [34]	Moderate	No rating	No rating	Weak	Weak	Moderate	Moderate	Moderate	Weak
24. Ferrazzi et al. (2001) [35]	Weak	No rating	No rating	No rating	Weak	Moderate	Strong	Strong	Weak
25. Okin et al. (2000) [57]	Weak	No rating	No rating	No rating	Weak	Moderate	Strong	Strong	Weak
26. Bates et al. (1999) [22]	Strong	Weak	Weak	Weak	Weak	Weak	Strong	Strong	Weak
27. Wickizer et al. (1998) [82]	Weak	No rating	Weak	Weak	Moderate	Moderate	Strong	Strong	Weak
28. Spillane et al. (1997) [74]	Strong	Weak	Weak	Strong	Weak	Weak	Moderate	Moderate	Weak
29. Bree et al. (1996) [23]	Strong	Weak	Moderate	Weak	Moderate	Moderate	Strong	Strong	Weak
30. Shea et al. (1995) [69]	Strong	Weak	Moderate	Weak	Strong	Weak	Strong	Strong	Weak
31. Cardiff et al. (1995) [26]	Moderate	No rating	Weak	Weak	Weak	Moderate	Strong	Moderate	Weak
32. Styrborn (1995) [76]	Strong	Weak	Strong	Strong	Weak	Moderate	Strong	Moderate	Weak
33. Rosenberg et al. (1995) [65]	Moderate	No rating	Moderate	Moderate	Moderate	Weak	Strong	Strong	Moderate
34. Jambunathan et al. (1995) [45]	Moderate	No rating	No rating	No rating	Weak	Weak	Moderate	Strong	Weak
35. Williams et al. (1994) [83]	Moderate	No rating	Weak	No rating	Weak	Weak	Moderate	Moderate	Weak
36. Wickizer (1992) [81]	Weak	No rating	Weak	Weak	Strong	Moderate	Strong	Moderate	Weak
37. Woodside et al. (1991) [84]	Moderate	No rating	Weak	Moderate	Weak	Weak	Weak	Moderate	Weak
38. Silver et al. (1992) [71]	Moderate	No rating	No rating	No rating	Weak	Weak	Weak	Weak	Weak
39. Fowkes et al. (1986) [36]	Strong	Weak	Weak	Weak	Weak	Weak	Weak	Weak	Weak
40. Echols et al.(1984) [32]	Weak	No rating	Weak	Weak	Weak	Moderate	Moderate	Moderate	Weak
41. Restuccia (1982) [63]	Strong	Weak	Weak	Weak	Strong	Moderate	Strong	Strong	Weak
42. Murphy (2014) [56]	Weak	No rating	Weak	Weak	Moderate	Moderate	Strong	Strong	Weak
43. Chiang et al. (2014) [27]	Weak	No rating	Weak	No rating	Weak	Moderate	Strong	Strong	Weak
44. Pillow et al. (2013) [60]	Weak	No rating	No rating	No rating	Weak	Moderate	Weak	Moderate	Weak
45. Dehaven et al. (2012) [31]	Moderate	No rating	Weak	No rating	Weak	Moderate	Strong	Strong	Weak
46. Tadros et al. (2012) [78]	Weak	No rating	No rating	No rating	Weak	Moderate	Strong	Strong	Weak
47. Shah et al. (2011) [68]	Strong	Weak	No rating	No rating	Strong	Moderate	Strong	Strong	Moderate
48. Stokes-Buzzelli et al. (2010) [75]	Weak	No rating	No rating	No rating	Weak	Moderate	Moderate	Strong	Weak
49. Grimmer- Somers et al. (2010) [38]	Weak	No rating	No rating	No rating	Moderate	Moderate	Moderate	Moderate	Weak
50. Grover et al. (2010) [39]	Weak	No rating	No rating	No rating	Weak	Moderate	Moderate	Strong	Weak
51. Skinner et al. (2009) [72]	Weak	No rating	No rating	No rating	Weak	Moderate	Moderate	Moderate	Weak
52. Shumway et al. (2008) [70]	Strong	Weak	Weak	Weak	Strong	Strong	Strong	Strong	Weak
53. Pope et al. (2000) [61]	Weak	No rating	Weak	Weak	Weak	Weak	Weak	Moderate	Weak
54. Moher et al. (1992) [55]	Strong	Weak	Strong	Strong	Weak	Weak	Strong	Strong	Weak
55. Kennedy et al. (1987) [47]	Strong	Strong	Strong	Strong	Weak	Strong	Weak	Weak	Weak
56. Kurant et al. (2018) [51]	Weak	No rating	No rating	No rating	Weak	Moderate	Weak	Moderate	Weak
57. Copeland et al. (2017) [28]	Weak	No rating	rating	No rating	Strong	Moderate	Strong	Moderate	Moderate
58. Pena et al. (2014) [58]	Weak	No rating	No rating	No rating	Weak	Moderate	Weak	Weak	Weak
59. Weilburg et al. (2017) [80]	Weak	No rating	No rating	No rating	Strong	Moderate	Strong	Strong	Moderate
60. Konger et al. (2016) [50]	Weak	No rating	No rating	No rating	Weak	Moderate	Weak	Moderate	Weak
61. El-Othmani et al. (2019) [33]	Moderate	No rating	No rating	No rating	Weak	Moderate	Weak	Moderate	Weak
62. Kim &Lee, (2020) [48]	Weak	No rating	Moderate	Moderate	Strong	Moderate	Strong	Strong	Moderate
63. Wasfy et al. (2019) [79]	Weak	No rating	No rating	No rating	Strong	Moderate	Strong	Strong	Moderate
64. Calsolaro et al. (2019) [25]	Weak	No rating	No rating	No rating	Moderate	Moderate	Strong	Strong	Moderate

## Abbreviations

ED: Emergency Department ED
LOS: Length of Hospital Stay
NCBA: Non-Controlled Before-and-After
